# Experimental Study on Ultrasonic Vibration-Assisted Grinding of SiCp/Al Composites Grinding

**DOI:** 10.3390/mi16030302

**Published:** 2025-03-04

**Authors:** Jinghao Jin, Jian Mao, Rong Wang, Mengyang Cui

**Affiliations:** 1 School of Mechanical and Automotive Engineering, Shanghai University of Engineering Science, Shanghai 201620, China; jjh19946297572@163.com (J.J.); wrong228@163.com (R.W.); mengyang198567@163.com (M.C.); 2 Key Laboratory of Intelligent Manufacturing Technology for Large Complex Thin-Walled Components of Aviation in Machinery Industry, Shanghai 201620, China; 3 Sichuan Research Institute, Shanghai Jiao Tong University, Chengdu 610213, China

**Keywords:** SiCp/Al composites, grinding force, ultrasonic vibration-assisted, grinding surface roughness

## Abstract

Aluminum matrix composites reinforced with silicon carbide particles (SiCp/Al) are widely used in aerospace fields with excellent properties, such as high specific strength, high specific stiffness, and high thermal conductivity. Due to the heterogeneous structure, its microstructure is one of the determinants of workpiece life, and ultrasonic vibration can improve the surface quality after grinding. Therefore, in this study, ultrasonic vibration-assisted grinding (UVAG) orthogonal tests were designed to study the surface morphology of SiCp/Al and the form of SiC particle removal under different machining parameters based on the SEM observation of the material surface, and to analyze the percentage of different kinds of grinding forces. The results show that the existence of particle fracture force depends on the relative sizes of the maximum undeformed chip thickness and the critical chip thickness. Through surface roughness testing and analysis, the influence of processing parameters on the surface roughness of the material is explored, and it is found that the application of ultrasound reduces the surface roughness of the material, which can be used as a guideline for the surface quality of the grinding process and the optimization of the process parameters.

## 1. Introduction

Metal matrix composites (MMCs) are engineered by embedding hard reinforcement materials, such as particles, fibers, or whiskers, into a ductile metallic matrix. This reinforcement enhances the interface between the matrix and the reinforcer, thereby facilitating the transfer of external loads to the metal matrix. Particle-reinforced metal matrix composites (PRMMCs) are particularly valued for their superior properties, including high specific strength, high specific stiffness, and excellent thermal conductivity [1]. Among these, SiC particle-reinforced aluminum matrix composites (SiCp/Al) stand out due to their combination of these desirable properties, along with enhanced wear resistance, a low coefficient of thermal expansion, and excellent dimensional stability. As a result, SiCp/Al composites are widely utilized in high-performance applications in industries such as aerospace, automotive, and manufacturing [2,3].

Grinding is typically the final process in the manufacturing of a machined part. However, in the case of SiCp/Al composites, conventional grinding methods often fail to meet the required processing standards due to the difference in material properties between the soft matrix and the hard, wear-resistant reinforcing particles [4]. Researchers have explored alternative machining techniques, such as laser machining. Although laser processing can achieve high material removal rates [5], the elevated temperatures generated during the process can adversely affect the material’s interior, making it unsuitable for applications involving cavity machining [6]. Water jet machining has also been investigated as a potential solution. Although water jets avoid the issues of macroscopic force and heat-affected zones, they are not suitable for precision machining [7]. Guo et al. [8] conducted a comprehensive investigation into the surface integrity and material removal mechanisms of silicon carbide particle-reinforced aluminum matrix composites (SiCp/Al composites) during high-speed grinding processes, with particular focus on elucidating the brittle-to-ductile transition occurring at critical grinding velocity.

Ultrasonic vibration-assisted grinding (UVAG) adds ultrasonic vibration to the tool to realize the periodic contact and separation between the tool and the workpiece, which improves the machining efficiency and the machined surface quality, and reduces the grinding force. The removal modes of SiCp/Al composites include brittle removal and plastic removal. Current research efforts remain insufficient in systematically investigating UVAG techniques [9,10], which demonstrate significant potential for enhancing both machining quality and processing efficiency. Moreover, the complex surface morphology generated by UVAG necessitates comprehensive characterization and validation to establish the rationality of surface integrity evaluation, presenting a critical knowledge gap requiring multi-parameter analysis beyond conventional assessment metrics [11]. Lin et al. [12] conducted cutting tests on SiCp/Al composites with a 20% volume fraction of SiC and observed that machining-induced stress concentrations near the particles often lead to microcrack formation on the shear surface. As cracks propagate, particle fragmentation occurs, resulting in discontinuous chips. The chip morphology was found to be dependent on the SiC particle volume fraction and cutting parameters. Gallab [13] investigated the turning of SiCp/Al composites with a 20% SiC volume fraction and examined the influence of cutting parameters on both the removal mode and surface quality. He found that SiC particles were primarily removed by debonding and crushing, leaving defects such as voids and microcracks on the aluminum matrix surface. Increased feed rates and depth of cut were found to promote chip chipping and discontinuous chip formation, which deteriorated surface quality. On the other hand, increasing cutting speed favored plastic removal and enhanced the material removal rate. Zhao et al. [14] conducted a single-grit scratch test to explore the material removal mechanism of SiCp/Al composites. Their findings indicated that the macroscopic removal process closely resembled that of the aluminum matrix, with both plowing and shear zones. The removal process involved a coupling of plastic removal of the aluminum matrix and brittle removal of SiC particles. The presence of SiC particles induced tearing and cracking of the matrix, while the aluminum matrix surrounding the SiC particles increased their plasticity, thus enhancing the likelihood of plastic removal of SiC particles. However, the particle removal mechanism remains primarily dependent on experimental observations and is difficult to predict and control, although surface quality can be managed by adjusting process parameters.

Due to defects such as pits being generated when SiC particles are removed, the surface roughness of SiCp/Al composites tends to be high after the grinding process. Yin et al. [15] established an analytical model for the grinding force of SiCp/Al composites and examined the surface morphology after grinding. Their study established a relationship between processing parameters and surface characteristics, and through parameter optimization, they achieved a surface roughness of 0.6 µm. Li et al. [16] investigated the correlation between grinding wheel wear and surface defects during the grinding process of particle-reinforced aluminum matrix composites, and they found that applying ultrasonic vibration assistance during grinding significantly improved the surface quality of the material. Feng et al. [17] conducted a comparison test between ultrasonic vibration-assisted scratching and normal scratching on SiCp/Al composites. Their results demonstrated that ultrasonic vibration-assisted scratching enhanced surface quality and minimized internal damage to the workpiece. Gu et al. [18] used ultrasonic vibration-assisted grinding to process SiCp/Al composites, and analyzed the resulting surface morphology, combined with the axial ultrasonic vibration influence coefficient, the grinding surface roughness model was constructed, and the maximum error of the model was 3.64%. Zheng et al. [19] investigated the surface formation mechanisms of conventional grinding and UVAG through finite element simulations. A comparative analysis of the two mechanisms was conducted to elucidate the advantages of UVAG in achieving superior surface quality. Building upon these findings, the surface characteristics of UAG were further analyzed. The surface morphology was systematically evaluated and validated using multiple metrics, including surface roughness, fractal dimension, skewness, and surface anisotropy. This multi-parameter approach addresses the limitations of relying solely on surface roughness or fractal dimension to characterize machined surfaces.

In this study, orthogonal tests were designed to investigate the grinding mechanism of SiCp/Al composites, the distribution of different grinding forces during the process was analyzed, and the mode of SiC particle removal was examined. The surface morphology and roughness of the material after grinding were evaluated. Additionally, the relationship between ultrasonic vibration parameters, machining parameters, and surface roughness was explored, with the aim of improving surface quality and identifying the optimal combination of machining parameters.

## 2. SiCp/Al Composite Grinding Test

### 2.1. Sample Material and Grinding Wheel Parameters

The research object is SiCp/2009Al with a volume fraction of 20%, and particle size ranging from 5 to 7 µm. The raw material is a plate with a size of 180 × 45 × 15 mm, and the heat treatment state is T4. The specimen was processed by wire cutting and then milled into a specimen with dimensions of 30 × 30 × 17 mm. The specific composition, content, and physical properties of the material are provided in Table 1 and Table 2. For pre-treatment, the material was subjected to milling using a cemented carbide cutter, diameter D = 10 mm, number of teeth Z = 4, milling cutter line speed is 125.66 mm/min, feed speed is 320 mm/min, milling depth is 0.4 mm.

The abrasive grains on the grinding wheel are randomly distributed, and the static abrasive density of the wheel is calculated as follows:

(1) $$C_{s}\left(Z\right)=A_{s}\cdot Z^{K_{s}}$$

where $A_{s}$ and $K_{s}$ depend on the parameters of the static abrasive grain density empirical function, the number of abrasives per unit area at different heights was measured using a super-depth-of-field 3D microscope and fitted accordingly. The values of (a) and (b), derived from the abrasive density fitting at different heights, are 22.1 and 0.0258, respectively. *Z* is the distance from the abrasive grain to the grinding wheel.

In this study, grinding tests were conducted using the ceramic-bonded CBN grinding wheel from San Yan Superhard Materials Co., Ltd, Zhengzhou, China. The abrasive grain morphology of the grinding wheel was observed using the KH-7700 electron microscope from HIROX Co., Ltd, Tokyo, Japan. Additionally, the distribution of various grinding wheel parameters is presented in Table 3. *r* is effective radius of abrasive grains, and *θ* is the cone angle of the grain, approximately 2 rad.

### 2.2. Ultrasonic Vibration-Assisted Grinding Test

**Figure 1:**
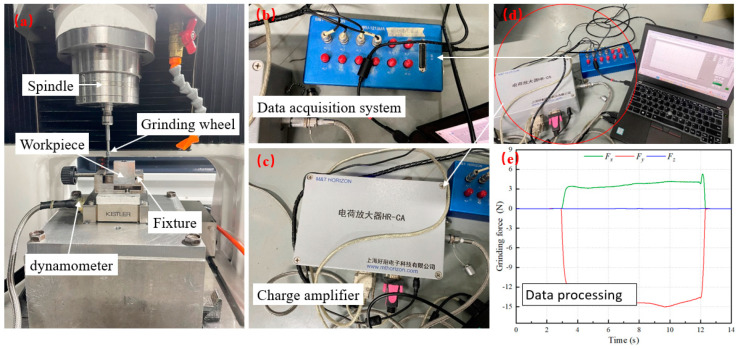
Ultrasonic vibration grinding test force measurement system. (**a**) grinding machining center; (**b**) grinding force data collector; (**c**) electric charge amplifier; (**d**) data receiving and processing systems; (**e**) force single processing.

UVAG experiments were conducted on a GJJG-XM30 high-precision ultrasonic grinding and compound machining center, utilizing a custom fixture to secure the workpiece material. The grinding force signal acquisition system consists of a dynamometer, charge amplifier, data acquisition system, and computer software as shown in Figure 1. The Kistler 9256C2 dynamometer from Switzerland was mounted on the table using an adapter plate, and the workpiece fixture was attached to the dynamometer, with the workpiece clamped in place. The force gauge is connected to the input of the charge amplifier, the data collector is connected to the output of the charge amplifier and the output of the data acquisition system is connected to the computer. The grinding force signals were processed using HRsoft_DW_V2.22 software.

The grinding force exhibits significant fluctuations during the initial and final stages, so the tangential force and normal grinding force at the stable grinding stage were selected for analysis. In order to eliminate the influence of spindle vibration on the grinding force, the Fourier transform is used to process the grinding force signals during both spindle idling and grinding. The amplitude-frequency signal during idling was found to be 400 Hz, while the amplitude-frequency signal during stable grinding was 125 Hz. Since the idling frequency exceeds the range of the dynamometer, the experimental data were processed by low-pass filtering. The low-pass cutoff frequency was set to 10 Hz, and the high-pass cutoff frequency was set to 150 Hz, which is above the frequency of the stable grinding phase.

In this study, a four-factor, three-level orthogonal test was designed. The four factors were: spindle speed n, feed speed $v_{w}$, grinding depth $a_{p}$, and air pressure P. The standard orthogonal test table of L9 (3, 4) was used for the design of the experimental program, and the detailed arrangement of the test program is provided in Table 4.

The surface of the workpiece was pre-grinded before the test to meet the straightness and roughness requirements for finish grinding, and the parameters of pre-grinding were n, 10,000 r/min; $V_{w}$, 200 mm/min; $a_{p}$, 30 µm; P, 0.5 MPa (equivalent to 0 amplitude). Orthogonal grinding tests were conducted on smooth surfaces. Before each test, the parallelism and straightness of the clamped workpiece were measured with a micrometer, and the straightness of the workpiece was maintained within 3 µm. In order to reduce the random error and improve the accuracy of the data, each group of tests was repeated three times, abnormal data were eliminated, and the average value was taken as the final test result.

### 2.3. Surface Morphology and Surface Roughness Testing of SiCp/Al Composites

After ultrasonic vibratory grinding, the surface of SiCp/Al composites was examined using the Sigma-300 scanning electron microscope (SEM) from Carl Zeiss AG, Germany, in secondary electron imaging mode, which has a resolution of 3.5 nm and a magnification range of up to 250,000 times. After SEM scanning, SiCp/Al composites produce more defects on the surface after grinding. These defects were categorized into four main types: SiC particles pressed into the matrix after debonding, SiC particles pulled out, SiC particles are broken, and Al matrix is coated. The specific forms of SiC particle removal were found to vary depending on the processing parameters, with the test results discussed in Section 3.

**Figure 2:**
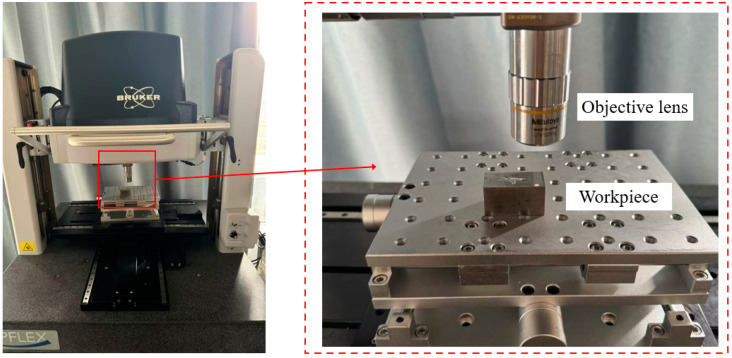
Npflex white light interference profiler.

Surface roughness testing was measured using the White Light Interferometer (model NPFlex) from Bruker Nano Surfaces Division, America, with specifications: 500 × 500 × 400 mm. The objective lens exhibits a vertical positioning accuracy of 0.1 nm, a maximum scanning range of 10 mm, and a minimum lateral resolution of 0.38 µm, and sub-nanometer display accuracy. The equipment device diagram shown in Figure 2. The device scans the surface of the object with white light, automatically performing calculations and generating roughness values. To isolate roughness from waviness, a Gaussian filter with a nesting index (cutoff wavelength) of 0.8 mm was applied to the raw surface data. Prior to roughness measurement, each grinding surface was cleaned using ultrasonic cleaning equipment to remove any remaining impurities. For each surface, five points were randomly selected for measurement, and the average value was recorded as the surface roughness of the grinding sample.

## 3. Results and Analysis

### 3.1. Analysis of UVAG Force and Removal Mechanism

The removal mechanism of SiC particles is closely related to the surface quality achieved after processing, with both brittle and plastic removal modes observed during the grinding process. Since SiC particles are brittle materials, based on the fracture mechanism of brittle materials, controlling the critical grinding depth can control the plastic removal of brittle materials. The condition for determining the brittle-to-plastic transition is based on the relative size of the maximum undeformed chip thickness and the critical chip thickness. When the maximum undeformed chip thickness is smaller than the critical chip thickness of the material, the brittle material is removed in a plastic manner. Conversely, when the maximum undeformed chip thickness exceeds the critical value, the material undergoes brittle removal. In this study, the indentation mechanics method is used to describe the composite material force, based on the Griffith fracture energy theory and the critical chip thickness model of brittle material without cracks constructed by Bifano et al. [20]. Wang et al. [21] found that the dynamic fracture toughness of brittle material is 0.3 times of the static one in dynamic machining, and the improved critical chip thickness model is calculated as:

(2) $$d_{c}=K\beta_{1}\left(\frac{E}{H_{v}}\right).{\left(\frac{K_{ID}}{H_{v}}\right)}^{2}$$

where E is Young’s modulus, $H_{v}$ is the hardness of the material, $K_{ID}$ is the dynamic fracture toughness of the material, $K_{ID}$ = 0.3$K_{IC}$, $K_{IC}$ is the static fracture toughness of the material, $\beta_{1}$ is the model parameter, which is 0.15, and K is the correction parameter considering the machining process.

The maximum undeformed chip thickness is calculated as follows:

(3) $$a_{m}=\sqrt{\frac{6a_{p}}{CN_{d}}\cdot \frac{v_{w}}{v_{s}}\cdot l_{u}^{-1}}$$

where $N_{d}$ is the number of abrasive grains, $l_{u}$ is the ultrasonic vibration arc length, and C is a model parameter.

Ultrasonic vibration-assisted grinding increases the contact arc length, resulting in a reduction in the maximum undeformed chip thickness. This facilitates a shift toward plastic removal compared to conventional grinding. When the maximum undeformed chip thickness is smaller than the critical chip thickness, the SiC particles are primarily removed plastically, and the SiCp/Al composites are subjected to grinding forces such as rubbing force, plowing force, and chip formation force. Conversely, when the maximum undeformed chip thickness exceeds the critical chip thickness, brittle fracture of SiC particles occurs, and the grinding forces also include particle fracture force, which increases the overall grinding force, which leads to an increase in the grinding force.

**Figure 3:**
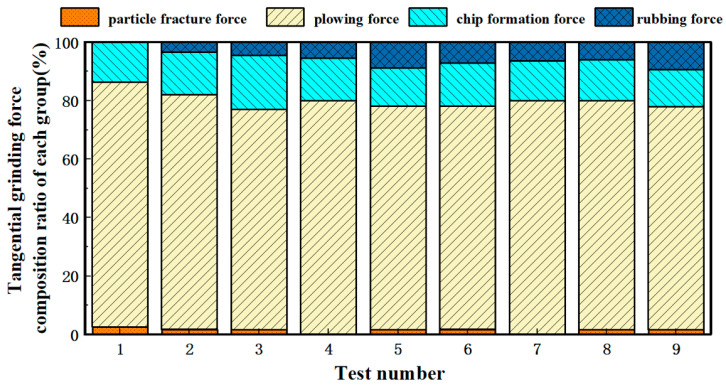
Composition of tangential grinding force for each group of specimens.

**Figure 4:**
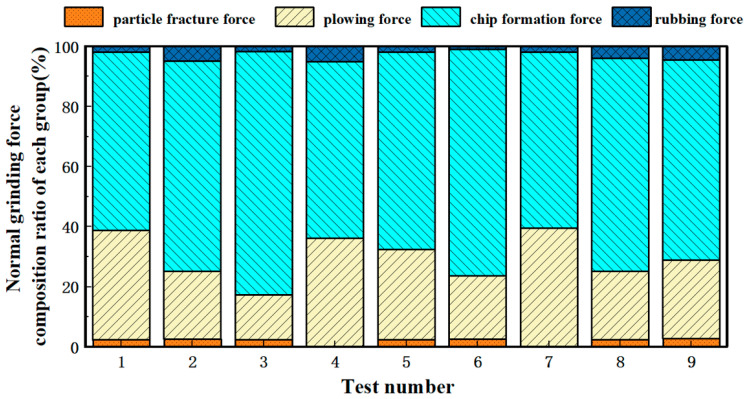
Composition of normal grinding force for each group of specimens.

Under different chip thickness conditions, the material removal mechanism was applied, with the process parameters substituted to obtain the fourth and seventh test groups where $a_{m}$ < $d_{c}$, indicating plastic removal without the involvement of particle fracture force. In contrast, for the remaining groups where $a_{m}$ > $d_{c}$, particle fracture force contributes to the grinding force, leading to brittle removal. The composition of ground forces in this section is derived from theoretical analysis based on an analytical model, while the specific material removal mechanisms occurring during the grinding process are discussed in Section 3.2 through experimental analysis utilizing SEM (scanning electron microscopy) imaging. The material removal mechanisms during grinding were evaluated based on the proposed formulations, with force component ratios simulated through MATLABR2023A software, as illustrated in Figure 3 and Figure 4. The results demonstrate that the plowing force dominates the tangential grinding force, while the chip formation force constitutes the primary component of the normal grinding force. This phenomenon may be attributed to the intensified abrasive wear caused by the presence of SiC particles. Furthermore, the substantial heat generation during grinding promotes adhesion between abrasives and workpiece surfaces. Such adhesive interactions reduce the effective grinding depth, shifting most surface material removal to a plowing mechanism rather than direct cutting. In the tangential direction, the plow phenomenon is the main removal method, which leads to an increase in the plowing force; in the normal direction, the diameter cutting is still the dominant removal method, with cutting and forming forces being the most significant contributors.

### 3.2. SEM Test Results and Analysis

**Figure 5:**
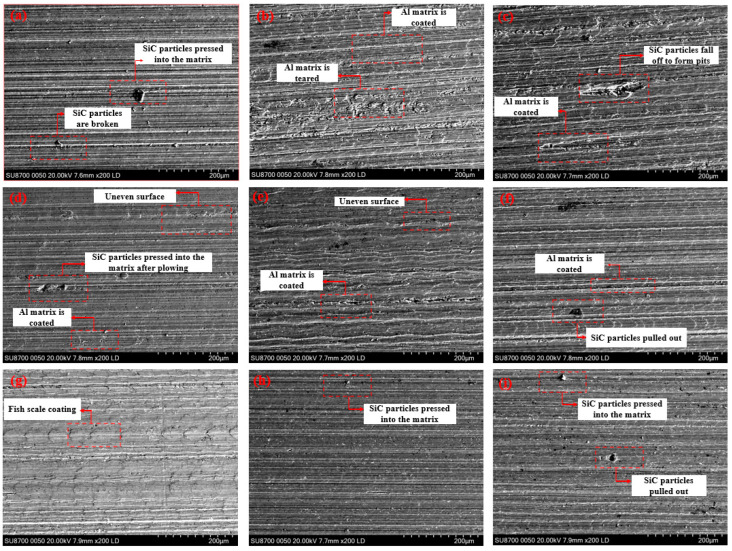
Workpiece of orthogonal test grinding surface topography workpiece, groups 1–9 corresponding to figures (**a**–**i**).

Figure 5 shows the observed surface morphology of each orthogonal test workpiece after ultrasonic vibration-assisted grinding. It can be seen that each workpiece surface presents a different surface morphology corresponding to the changes in processing and ultrasonic vibration parameters. It is observed that the surface of specimen 2 is scratched by a large pit, indicating that SiC particles were pulled out during grinding. The ejected particles were subsequently pushed forward by the grinding tool, leaving scratches on the surface. Specimens 1, 4, and 9 have raised particles on the surface and show more regular plastic scratches near the particles, which were pulled out and then pressed into the aluminum matrix by the tool. The surfaces of specimens 5, 6, and 7 are coated areas, proving that at this time the surface of the workpiece was burned, at this time, generated a large grinding heat. Specifically, specimen 7 does not show obvious defects, which suggests that plastic removal of SiC particles occurred at this condition. This observation aligns with the theoretical analysis in Section 3.1, confirming the plastic removal mechanism. When SiC particles are removed plastically, the surface quality of the workpiece improves.

A comparison of specimens 1 and 7 reveals that under identical grinding depth and ultrasonic amplitude conditions, the surface quality of specimen 7 is superior. This can be attributed to the higher grinding wheel rotational speed of specimen 7, which generates more grinding heat. Although it will lead to defects in the coating of the base material, the temperature increase will lead to a larger strain. When the strain reaches the recrystallization critical strain, grain refinement occurs, enhancing the interface strength between the phases. Consequently, fewer defects such as grain pull-out or indentation are observed, resulting in improved surface quality. It should be noted that surface quality is not inversely proportional to ultrasonic vibration. While ultrasonic vibration is not the sole factor influencing surface quality, higher ultrasonic amplitudes generally yield better surface quality. This improvement may be attributed to the grain refinement promoted by ultrasonic vibration, which also strengthens the interface between the two phases. And it also enhances the strength of the interface between the two phases to reduce the formation of defects, such as particle dislodgement pits, so that the surface quality is better. 

### 3.3. Roughness Results and Analysis

**Figure 6:**
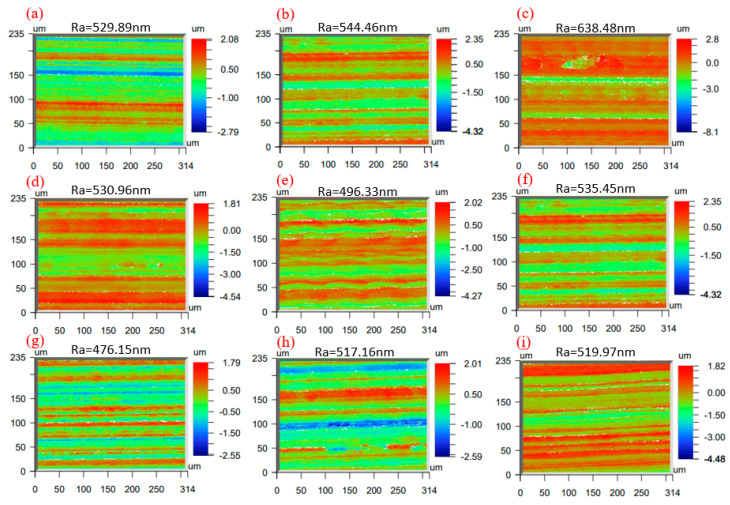
The measurement results of the roughness of the ground surface, groups 1–9 workpieces corresponding to (**a**–**i**).

Prior to roughness measurement, an ultrasonic cleaning device was used to clean the grinding surface in order to remove debris and other impurities generated during the machining process. Roughness was measured at five different locations on each workpiece, and the average value of Ra was taken as the surface roughness of the grinding surface; the surface roughness values obtained from the orthogonal grinding test are shown in Figure 6. The relationship between the process parameters and surface roughness can be established. By comparing the data from groups (a), (b), (c), (g), (h), and (i) in Figure 6, it is evident that the overall surface roughness in experiments 7–9 is significantly lower than that in experiments 1–3. This suggests that an increase in spindle speed leads to a gradual reduction in surface roughness. The reasons are as follows: the increase in grinding wheel rotational speed leads to more frequency of contact between abrasive grains and workpiece per unit time, which strengthens the cutting action and makes the surface smoother after grinding, and the increase in spindle rotational speed can improve the material removal rate. Concurrently, at elevated speeds, the ultrasonic vibration effect on the abrasive grain path becomes more pronounced, reducing the grinding force’s impact and further improving surface quality.

A horizontal comparison of data within the same row in Figure 6 reveals that, except for groups (d) and (e), an increase in feed rate consistently elevates surface roughness. While the results of groups 4 and 5 may be influenced by interaction effects such as ultrasonic air pressure, the general trend demonstrates a positive correlation between surface roughness and feed rate. This is primarily due to the presence of SiC particles, which intensify abrasive grain wear and result in greater abrasive grain loss. The higher feed speed will increase the frequency of grinding contact, causing the abrasive grains to become more irregular and resulting in higher surface roughness. Furthermore, the abrasive grains are continuously obstructed by the workpiece, and the increase in the feed speed will increase the obstruction effect, limiting the material’s plastic flow and reducing the efficiency of SiC particle removal. Consequently, the surface quality deteriorates.

The range analysis in Table 5 indicates that surface roughness increases with grinding depth. However, due to the relatively weaker influence of grinding depth compared to the aforementioned factors, the correlation between grinding depth and roughness cannot be directly discerned. This is due to the reduced heat dissipation capability in the contact area, which leads to a buildup of heat in the grinding zone. The accumulated heat raises the temperature of the grinding area, causing phenomena such as substrate coating, which increases the surface roughness. Additionally, the elevated temperature makes the strength of the interface between the two phases higher, which also makes the surface roughness larger.

Analyzing the effect of ultrasound on the grinding surface roughness, it can be found that ultrasonic vibration can reduce the surface roughness to a certain extent. However, the surface roughness at an air pressure of 0.65 MPa and ultrasonic amplitude is generally higher than that at 0.6 MPa, which indicates that the ultrasonic amplitude does not exhibit a simple linear relationship with surface roughness. When the air pressure is lower, the tool may experience an extrusion effect when in contact with the workpiece under the same machining parameters, leading to the formation of pits on the material surface and resulting in poor surface quality. After adding ultrasonic vibration, there is more hammering contact between the tool and the material, which can break some SiC particles, thus reducing the formation of crater defects and improving the surface quality. However, excessively high ultrasonic amplitude may cause the tool to impact the substrate surface directly, resulting in defects appearing directly on the surface of the workpiece and an increase in surface roughness. Therefore, an optimal ultrasonic amplitude should be selected to achieve the best surface quality after grinding.

The range analysis results of the orthogonal experiment are summarized in Table 5. The average value 1 in the spindle speed column indicates the mean surface roughness value under the first-level spindle speed parameter (8000 r/min) in the orthogonal experiment, which is derived from three experimental measurements. Other average values should be interpreted similarly. Through subsequent range analysis, the range magnitude can be obtained, thereby enabling determination of the influence degree of different machining parameters on surface roughness values. As evident from the data, spindle speed demonstrated the most pronounced influence on surface roughness, while grinding depth exhibited the minimal effect, indicating that spindle speed is the dominant parameter governing surface quality. The order of significance for processing parameters affecting surface roughness was determined as follows: n > $v_{w}$ > P > $a_{p}$. Notably, $v_{w}$ and P demonstrated comparable influence magnitudes. Consequently, to achieve superior surface quality, it is recommended to prioritize higher spindle speeds, reduced feed rates, and increased air pressure during machining operations.

Due to the inherent limitations of orthogonal experimental design, this study did not thoroughly investigate the effects of multi-parameter interactions on surface roughness. Future research could incorporate additional experimental points, to develop interaction models among parameters, perform optimization, and identify optimal process parameters. This direction will be critical for advancing subsequent studies.

## 4. Conclusions

In this study, the grinding mechanism and surface quality of ultrasonic vibration-assisted grinding of SiCp/Al composites are investigated through experiments. It was found that plastic removal of SiC particles improves the surface quality of the material. The main conclusions are as follows:

(1) The maximum chip thickness and critical undeformed chip thickness models were established, and the removal mechanism of SiC particles under various processing parameters was examined to determine the conditions for plastic removal of SiC particles. The distribution of different grinding forces during the process was analyzed, revealing that the plowing force accounted for the largest proportion of the tangential grinding force, while the chip formation force contributed the most to the normal grinding force.
(2) Surface defects of SiCp/Al composites after grinding were observed using scanning electron microscopy (SEM). These defects included debonding of SiC particles, SiC particles being pressed into the substrate, SiC particles being pulled out to form craters, surface scratches caused by abraded SiC particles or the grinding tool, and substrate coating defects due to high temperatures in the machining zone. It was concluded that surface quality can be effectively controlled by adjusting the machining parameters.
(3) The surface roughness of SiCp/Al composites was measured using a white light interference profilometer. The results showed that the surface roughness of the material decreased with the increase in the spindle speed and increased with the increase in the feed rate. The effect of ultrasonic vibration on the roughness is not unidirectional. As ultrasonic amplitude increased, surface roughness initially decreased and then increased. This behavior is attributed to the fact that ultrasonic vibration primarily promotes fracture removal of SiC particles, reducing the formation of craters from pulled-out particles and scratches caused by the grinding tool. However, excessive amplitude can lead to tool-induced scratches, negatively impacting surface quality.

## Figures and Tables

**Table 1:** Chemical composition of 20% SiCp/2009Al composites (%, mass fraction).

SiC	Mg	Si	Ti	Fe	Cu	Al
20	1.25	0.22	<0.03	0.08	3.36	Bal.

**Table 2:** Material properties of 20% SiCp/2009Al composites (T4 state).

Tensile Strength MPa	Yield Strength MPa	Elongation %	Density g/cm^3^	Young’s Modulus GPa	Poisson’s Ratio	Coefficient of Thermal Conductivity W·m^−1^·K^−1^	Coefficient of Linear Expansion (20–100 °C) 10^−6^·°C^−1^
560	370	8.5	2.84	105	0.38	178	15.1

**Table 3:** The parameters of the wheel.

*θ*	*r* (µm)	*A_s_*	*K_s_*
114.6	60.25	22.1	0.0258

**Table 4:** Grinding test program table.

Test Number	Spindle Speed *n* (r/min)	Feed Speed *v_w_* (mm/min)	Grinding Depth *a_p_* (µm)	Air Pressure *P* (MPa)
1	8000	100	10	0.65
2	8000	200	20	0.6
3	8000	300	30	0.55
4	10,000	100	20	0.55
5	10,000	200	30	0.65
6	10,000	300	10	0.6
7	12,000	100	30	0.6
8	12,000	200	10	0.55
9	12,000	300	20	0.65

**Table 5:** Surface roughness range analysis results (nm).

	Spindle Speed n	$\mathrm{Feed}\;\mathrm{Speed}\;v_{w}$	$\mathrm{Grinding}\;\mathrm{Depth}\;a_{p}$	Air Pressure P
Repeat Number	3	3	3	3
Average value 1	570.94	512.33	527.50	562.20
Average value 2	520.91	519.32	531.80	518.69
Average value 3	504.43	564.63	536.99	515.40
Range	66.51	52.30	9.49	46.80

## Data Availability

The original contributions presented in this study are included in the article. Further inquiries can be directed to the corresponding author.
